# Resistance patterns and transmission of mono- and polyresistant TB: clinical impact of WGS

**DOI:** 10.1093/jacamr/dlad108

**Published:** 2023-10-04

**Authors:** Matúš Dohál, Věra Dvořáková, Miluše Šperková, Martina Pinková, Andrea Spitaleri, Erik Michael Rasmussen, Mária Škereňová, Michaela Krivošová, Eduard Gondáš, Igor Porvazník, Ivan Solovič, Daniela Maria Cirillo, Juraj Mokrý

**Affiliations:** Biomedical Centre Martin, Jessenius Faculty of Medicine in Martin, Comenius University, Bratislava, Slovakia; National Reference Laboratory for Mycobacteria, National Institute of Public Health, Prague, Czech Republic; National Reference Laboratory for Mycobacteria, National Institute of Public Health, Prague, Czech Republic; National Reference Laboratory for Mycobacteria, National Institute of Public Health, Prague, Czech Republic; Emerging Bacterial Pathogens Unit, Division of Immunology, Transplantation and Infectious Diseases, IRCCS San Raffaele Scientific Institute, Milan, Italy; Vita-Salute San Raffaele University, Milan, Italy; International Reference Laboratory of Mycobacteriology, Statens Serum Institut, Copenhagen, Denmark; Biomedical Centre Martin, Jessenius Faculty of Medicine in Martin, Comenius University, Bratislava, Slovakia; Department of Molecular Medicine, Jessenius Faculty of Medicine in Martin, Comenius University, Bratislava, Slovakia; Biomedical Centre Martin, Jessenius Faculty of Medicine in Martin, Comenius University, Bratislava, Slovakia; Biomedical Centre Martin, Jessenius Faculty of Medicine in Martin, Comenius University, Bratislava, Slovakia; Department of Clinical Microbiology and Department of Pneumophthiology, National Institute of Tuberculosis, Lung Diseases and Thoracic Surgery, Vyšné Hágy, Slovakia; Faculty of Health, Catholic University, Ružomberok, Slovakia; Department of Clinical Microbiology and Department of Pneumophthiology, National Institute of Tuberculosis, Lung Diseases and Thoracic Surgery, Vyšné Hágy, Slovakia; Emerging Bacterial Pathogens Unit, Division of Immunology, Transplantation and Infectious Diseases, IRCCS San Raffaele Scientific Institute, Milan, Italy; Department of Pharmacology, Jessenius Faculty of Medicine in Martin, Comenius University, Bratislava, Slovakia

## Abstract

**Objectives:**

Rapidly diagnosing drug-resistant TB is crucial for improving treatment and transmission control. WGS is becoming increasingly accessible and has added value to the diagnosis and treatment of TB. The aim of the study was to perform WGS to determine the rate of false-positive results of phenotypic drug susceptibility testing (pDST) and characterize the molecular mechanisms of resistance and transmission of mono- and polyresistant *Mycobacterium (M.) tuberculosis*.

**Methods:**

WGS was performed on 53 monoresistant and 25 polyresistant *M. tuberculosis* isolates characterized by pDST. Sequencing data were bioinformatically processed to infer mutations encoding resistance and determine the origin of resistance and phylogenetic relationship between isolates studied.

**Results:**

The data showed the variable sensitivity and specificity of WGS in comparison with pDST as the gold standard: isoniazid 92.7% and 92.3%; streptomycin 41.9% and 100.0%; pyrazinamide 15% and 94.8%; and ethambutol 75.0% and 98.6%, respectively. We found novel mutations encoding resistance to streptomycin (in *gidB*) and pyrazinamide (in *kefB*). Most isolates belonged to lineage 4 (80.1%) and the overall clustering rate was 11.5%. We observed lineage-specific gene variations encoding resistance to streptomycin and pyrazinamide.

**Conclusions:**

This study highlights the clinical potential of WGS in ruling out false-positive drug resistance following phenotypic or genetic drug testing, and recommend this technology together with the WHO catalogue in designing an optimal individualized treatment regimen and preventing the development of MDR TB. Our results suggest that resistance is primarily developed through spontaneous mutations or selective pressure.

## Introduction

Nearly 1.5 million people die from TB annually, which is still among the deadliest infectious diseases worldwide.^1^ The eradication of TB by 2035, as proposed by the WHO, is complicated by the emergence and global spread of drug-resistant (DR) TB. The overall proportion of MDR TB (i.e. TB caused by rifampicin- and isoniazid-resistant strains) is approximately 3%–4% in newly diagnosed patients and 18%–21% in prior-treatment patients. In addition, the prevalence of monoresistant (resistance to a single first-line drug) and polyresistant (resistance to two or more first-line drugs but not to both isoniazid and rifampicin) strains is almost 17%.^2^ Improper management of mono- and polyresistant TB may contribute to acquiring further drug resistance and evolving MDR TB, thus emphasizing the importance of prompt diagnostics and determination of the resistance profile in clinical settings.^3^

Previous studies showed that susceptibility testing by conventional phenotypic and genotypic methods (Xpert MTB/RIF) can be associated with false-positive resistance or false-negative susceptibility, and can lead to mismanagement of patients’ treatment regimens.^4,5^ Therefore, the WHO recommends the sequencing approach as a suitable alternative for detecting resistant forms of TB.^6^ WGS can simultaneously provide information on resistance to first-line and second-line drugs (including new-generation drugs) and information necessary for a detailed epidemiological investigation.^7^ Many studies have focused on WGS and the characterization of MDR-TB or rifampicin-resistant (RR) strains and demonstrated excellent predictive values of WGS in the *Mycobacterium tuberculosis* resistome.^7,8^ Despite the importance of the issue, we did not find any studies evaluating the utility of WGS to characterize mono- and polyresistant TB.

In this study, we performed WGS to diagnose mono- and polyresistant TB (except RR) and highlighted the potential of this technology to provide individualized treatment guidelines compared with phenotypic drug susceptibility testing (pDST).

## Materials and methods

### M. tuberculosis clinical isolates

We selected all monoresistant (except rifampicin) and polyresistant *M. tuberculosis* strains (*n* = 92) collected in the National Reference Laboratory for Mycobacteria in the Czech Republic and the National Reference Mycobacteriology Laboratory in Slovakia during the years 2018–20. *M. tuberculosis* isolates were retrieved from frozen archived collections stored at −80°C and recovered using the BACTEC Mycobacteria Growth Indicator Tube (MGIT) 960 system (Becton Dickinson, USA).

### pDST

pDST was performed on Löwenstein–Jensen (LJ) medium for all drugs; for pyrazinamide, we used the BACTEC MGIT 960 system. The media contained drugs at critical concentrations (CCs) recommended by the WHO: rifampicin (40.0 mg/L), isoniazid (0.2 mg/L), ethambutol (2.0 mg/L), pyrazinamide (100.0 mg/L), streptomycin (4.0 mg/L), kanamycin (30.0 mg/L), amikacin (30 mg/L), ethionamide (40.0 mg/L), cycloserine (40.0 mg/L), para-aminosalicylic acid (1.0 mg/L), moxifloxacin (1.0 mg/L), levofloxacin (2.0 mg/L), delamanid (0.016 mg/L), bedaquiline (0.25 mg/L) and linezolid (1.0 mg/L).^6^

To study the clinical relevance of mutations encoding resistance, we also used concentrations above and below the CC of each drug (Table 1).

**Table 1.** Concentrations of anti-TB drugs used in pDST

Drug		CC (mg/L)	
Isoniazid	0.1	0.2	1
Rifampicin	20	40	50
Ethambutol	1	2	5
Pyrazinamide	100	400	—
Streptomycin	1	4	10
Kanamycin	20	30	—
Amikacin	20	30	60
Ethionamide	20	40	—
Cycloserine	20	40	—
Para-aminosalicylic acid	0.5	1	—
Moxifloxacin	0.1	1	4
Levofloxacin	1	2	5
Delamanid	0.008	0.016	0.032
Bedaquiline	0.1	0.25	1
Linezolid	0.5	1	2

### Genomic DNA extraction and WGS

*M. tuberculosis* isolates were cultured using the BACTEC MGIT 960 system. DNA was extracted from 1 mL of heat-inactivated (95°C for 30 min in the biosafety level 3 laboratory) early-positive culture using the QIAamp DNA Mini Kit (QIAGEN, Hilden, Germany). DNA concentration was quantified by Qubit 4 technology using the Qubit dsDNA HS Assay Kit (Thermo Fisher Scientific, Waltham, USA) and normalized to 0.2 ng/μL to be used as input for the Nextera XT library preparation kit (Illumina, San Diego, CA, USA). Library preparation was performed according to manufacturer’s instructions. Libraries were batched and sequenced with an Illumina MiSeq (Illumina) using 2 × 150 bp paired-end chemistry.

### Bioinformatics processing of sequencing data

The sequences were analysed using the automated pipeline MTBseq (https://github.com/ngs-fzb/MTBseq_source) for detection of resistance mediating and phylogenetic variants and in order to study the agreement between phenotypic resistance to the different drugs, including isoniazid, cycloserine, ethambutol, streptomycin, and the genomic data.^9^ The mutations conferring resistance to the aforementioned drugs were identified by the WHO catalogue Excel file using in-house python scripting analysis.^10^ Using the concatenated sequence alignments, isolates were grouped by cluster analysis performed on the distance matrix generated by the MTBseq pipeline using in-house python script exploiting Matplotlib 3.3.2 and SciPy 1.5.2 version python libraries. The distance matrix was analysed using the hierarchical linkage clustering method with the nearest point algorithm using 5 SNPs as cut-off. The maximum parsimony phylogenetic tree and minimum spanning tree (MST) were constructed on 671 229 SNP positions of 78 clinical isolates, using RAxML version 8.2.12 and GrapeTree version 1.5.0 software, respectively.^11^ The phylogenetic tree was built with 100 bootstraps using the ‘GTRGAMMA’ model and visualized using iTOL web version 1.0.^12^ The maximum distance threshold of 5 SNPs was used for linked transmission.^13,14^ The tanglegram was calculated using dendextend R function version 1.15.1.^15^

### Ethics

This study was approved by the Ethics Committee of Jessenius Faculty of Medicine in Martin, Comenius University in Bratislava, Slovakia (EK 72/2018).

## Results

### M. tuberculosis strain characteristics

Of the 92 isolates collected between the years 2018 and 2020, 78 (84.8%) were successfully cultured and had interpretable pDST results. Complete phenotypic resistance profiles are shown in Table 2. All RR isolates were also MDR and were excluded from this study.

**Table 2.** Overview of isolates based on pDST results

Monoresistant		Polyresistant	
Drug	Number of isolates	Drugs	Number of isolates
INH	15	INH + STM	17
STM	20	INH + STM + PZA	1
		INH + STM + EMB	2
PZA	18	INH + STM + CS	1
		INH + STM + EMB + PZA	1
PZA^R^	3	STM + EMB	1
		STM + PZA	1

INH, isoniazid; PZA, pyrazinamide; STM, streptomycin; EMB, ethambutol; CS, cycloserine; PZA^R^, repeated pDST.

### Sequencing data quality

The minimum criteria for sequencing data quality were: mean coverage ≥30 and mapped reads ≥90% of the reference genome covered by the sequence read. These requirements were met for all samples, as the average depth of coverage was 52× and mapped reads 98.5%.

### Analysis of genotypic and phenotypic resistance to anti-TB drugs

Of the 78 *M. tuberculosis* phenotypically resistant isolates sequenced, 43 (57.7%) harboured at least one mutation associated with resistance to any anti-TB drug based on the WHO catalogue, indicating a high rate of phenotypically resistant strains without any mutation encoding resistance.^10^ Variable sensitivities and specificities were obtained for the genotypic–phenotypic correlation of resistance predictions: isoniazid (92.7% and 92.3%, respectively); ethambutol (75.0% and 98.6%); streptomycin (41.9% and 100.0%); and pyrazinamide (15.00% and 94.8%).

Mutations associated with resistance to isoniazid were predominantly found in the *katG* (S315T; 33/38) gene, followed by mutations in *inhA* (S94A; 2/38) and *fabG* (15C > T; 11/38) genes.

Six isolates had pyrazinamide resistance-conferring mutations in the *pncA* gene (A134V and W119C), although four isolates were susceptible to pyrazinamide according to pDST (exhibiting only resistance to isoniazid). In three phenotypic pyrazinamide-monoresistant isolates, we detected the mutation in *pncA* (G162R) and *kefB* (A107V), classified in ‘Group 3: uncertain significance’ based on the WHO catalogue. Variant calling analysis also revealed mutations at codon 87 (T87M) of the *pncA* in two pyrazinamide-monoresistant strains. Based on the low sensitivity of WGS, we repeated pDST against pyrazinamide in all previously phenotypically resistant *M. tuberculosis* isolates (*n* = 18) and confirmed pyrazinamide resistance in only 3/18, indicating that 83.3% of isolates were initially misdiagnosed.

The common mutations associated with resistance to streptomycin were at codons 43 (13/18) and 88 (3/18) in the *rpsL* gene. The other mutations were in the *rrs* and *gidB* genes. For phenotypic-resistant isolates with no known resistance genetic mutations (*n* = 25) identified, we assessed the other mutations in *gidB*, *rrs* and *rpsL* genes identified by variant calling analysis compared with mutation included in WHO catalogue. Fifteen (60%) of 25 isolates harboured the variants classified in ‘Group 3: uncertain significance’ (Table 3). Moreover, two novel frameshift potentially resistance-encoding mutations (489_489del; 355_356del) in the *gibB* gene were also found. Eight isolates did not show any mutation encoding resistance to streptomycin or showed variants classified in ‘Group 5: not associated with resistance’ in the WHO catalogue. To determine the resistance level, we tested three different concentrations of streptomycin (1, 4 and 10 mg/L) and observed distinct growth profiles across the isolates without a known mutation encoding resistance. Twelve of these isolates showed a reduced growth rate in a medium containing streptomycin at the CC (4 mg/L), 10 isolates exhibited normal growth, and 3 isolates were also resistant to streptomycin at a concentration above the CC (10 mg/L). Isolates with MICs above the CC, for which WGS did not identify any resistance-conferring mutations based on the WHO mutation catalogue, may be considered as falsely susceptible by genotypic drug susceptibility testing.

**Table 3.** List of mutations associated with resistance to streptomycin assigned to ‘Group 3: uncertain significance’ in WHO catalogue and their relationship to the extent of growth at different concentrations of streptomycin in phenotypically resistant and susceptible isolates

Mutation	Number of phenotypically resistant isolates (*n* = 17)	Number of phenotypically susceptible isolates (*n* = 4)	STM (1 mg/L)	STM (4 mg/L)	STM (10 mg/L)
*gidB* G34E	1	0	+++	+++	−
*gidB* L35P	3	0	+++/+++/+++	+++/+/+	−
*gidB* a-60c	3	0	+++/+++/+++	+++/+/+	−
*gidB* c-11t	2	0	+++	+++	−
*gidB* G157R	1	0	+++	+++	−
*gidB* A119D	1	0	+++	+++	−
*gidB* V202A	1	0	+++	+	−
*gidB* V77A	1	0	+++	+++	−
*gidB* L79W	0	1	−	−	−
*whiB6* c-82t	0	1	−	−	−
*whiB6* T51P**^a^**	2	0	+++/+++	+++/+++	−
*rrs* a-198c	1	0	+++	+	−
*rrs* c-1443g	0	1	−	−	−
*rrs* t-202a	0	1	−	−	−
*rrs* a514c**^a^**	1	0	+++	+++	−

STM, streptomycin. Extent of growth: +++, heavy growth; +, reduced growth; −, no growth.

a Mutation present with another mutation confirming resistance to STM.

For ethambutol, four isolates had resistance-conferring mutations in *embB*, the most frequent being M306I (3/4).

Three isolates phenotypically classified as polyresistant should be reclassified as MDR based on WGS results due to variants associated with rifampicin resistance identified in the *rpoB* gene (I491P; L430P; L452P).

For second-line anti-TB drugs tested, all isolates were phenotypically susceptible. One isolate was found with the mutation D461N in the *gyrA* gene, mediating resistance to levofloxacin and moxifloxacin. We also identified several mutations with uncertain significance in resistance to bedaquiline, delamanid and linezolid.

### Phylogenic analysis and association of specific lineage with a resistance profile

**Figure 1.**
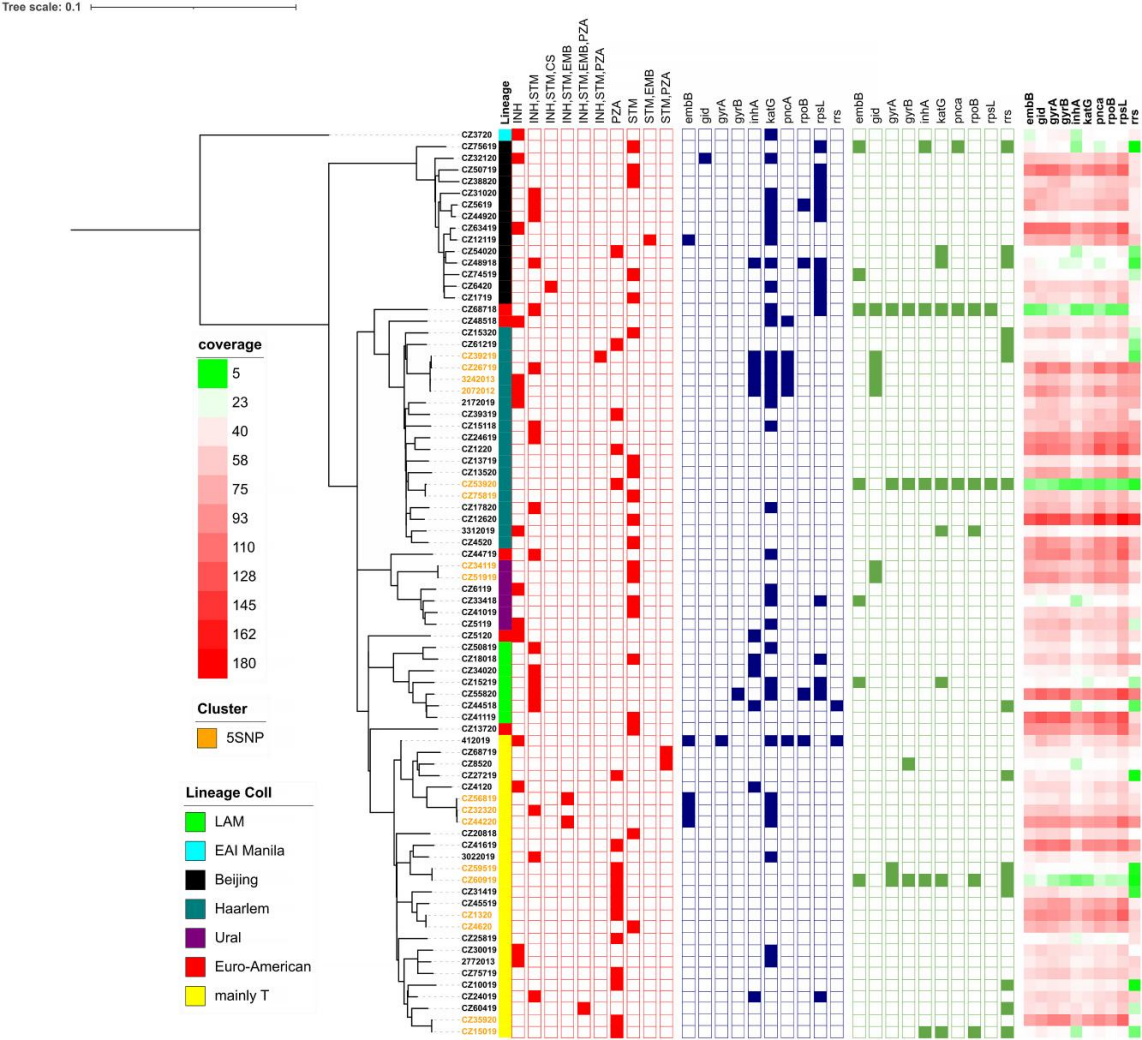
Phylogenetic tree of *M. tuberculosis* strains (*n* = 78), with their lineages, phenotypic drug resistance profiles, and mutations in genes encoding resistance. Isolates highlighted in orange are clustered within five SNPs. The first vertical band denotes the lineage. The red squares show the results of phenotypic testing, with filled squares representing resistance to the drug. The filled blue squares show the presence of genes associated with resistance. A filled green square means that the gene is not covered 100% at 8 ×  at least (indicating putative deletion of the gene). The coverage heatmap shows the average number of reads that cover the respective gene.

Phylogenetic lineages were inferred based on SNPs specific for certain *M. tuberculosis* sublineages based on a recently introduced SNP barcode classification.^16^ Samples with lower sequencing coverage were also included in the phylogenetic analysis, as the distance matrix was not affected (Figure S1, available as Supplementary data at *JAC-AMR* Online). The isolates were distributed within three major phylogenetic lineages: 63 isolates (80.1%) belonged to lineage 4; 14 isolates (17.9%) belonged to lineage 2 (14/14 lineage 2.2.1); and 1 isolate (2.0%) belonged to lineage 1 (for a more detailed sublineage classification see Figure 1). There were five subsets of Beijing strains. The majority (7/14) belonged to the Central Asia sublineage. Other Beijing sublineages were Asia/Africa 1 (1/14), Asian/Africa 2 (2/14), Europe/Russian W148 Outbreak (2/14) and Ancestral 3 (1/14).

We found several potential associations between phylogenetic lineage and resistance profile (Figure 1). Streptomycin resistance was predominantly encoded by the *rpsL* L43A mutation (13/18). The frequency of these mutations was highest in the Beijing lineage 2.2.1 (8/13). Surprisingly, all 23 (100%) isolates phenotypically resistant to streptomycin without a known resistance mechanism were identified within lineage 4. Among the 16 isolates phenotypically resistant to pyrazinamide without a known mutation, 15 (93.6%) belonged to lineage 4 (of which 9 isolates belonged to superlineage 4.8). Also, most isolates (66.6%) with mutations encoding resistance to pyrazinamide belonged to lineage 4.1.2.1 (Figure 1).

### Transmission of mono- and polyresistant TB

**Figure 2.**
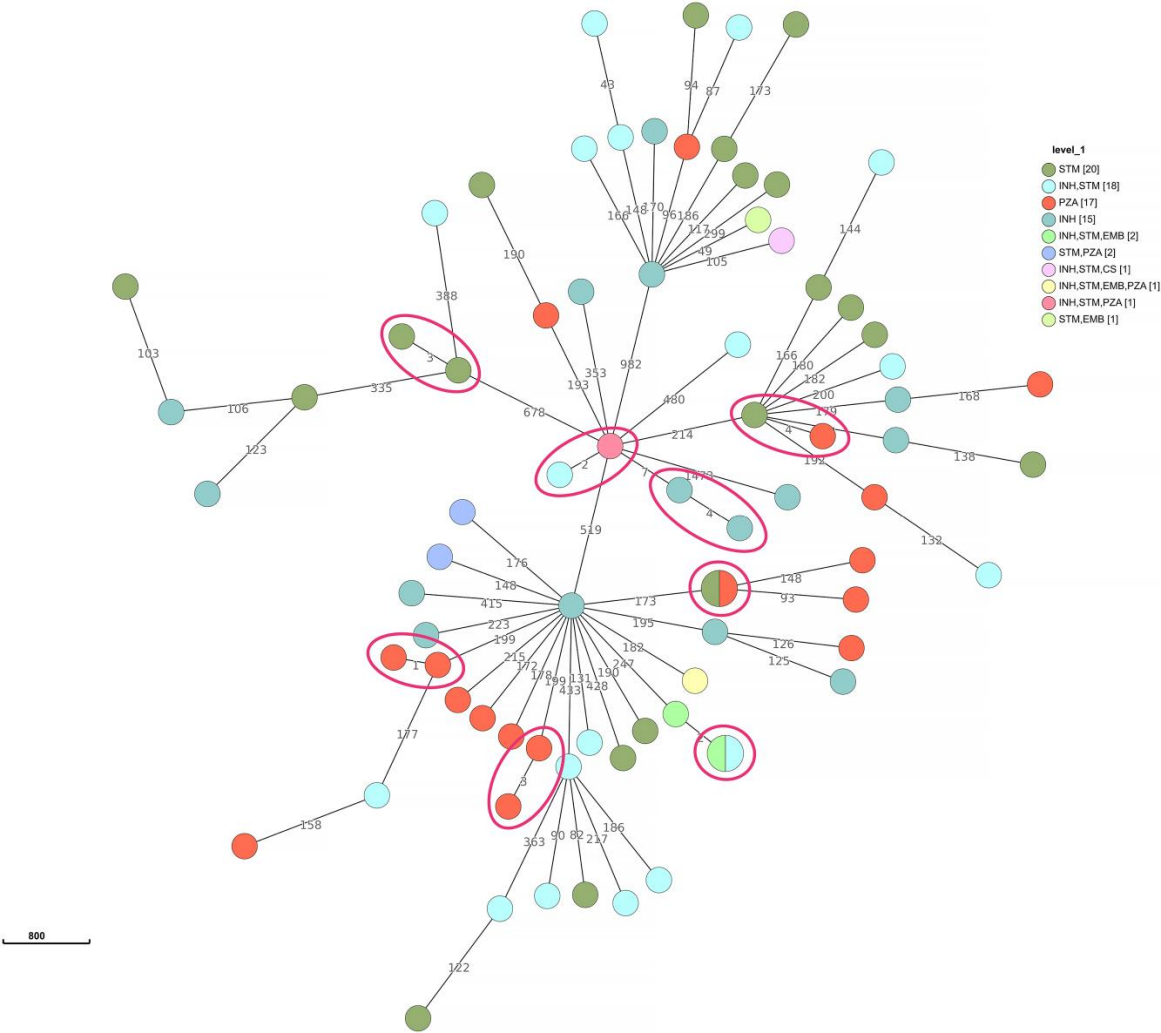
MST based on SNP differences between the strains, including the mono- and polyresistant strains collected in the Czech Republic and Slovakia. The maximum distance was set to five SNPs for linked transmission. Dot colouring indicates the resistance profile based on pDST.

Potential transmission clusters were identified by determining the pairwise SNP distance between the 78 mono- and polyresistant *M. tuberculosis* isolates. The distance ranged from 0 to 1473 SNPs. MST analysis with a predefined maximum cut-off of five SNP differences revealed eight clusters (Figure 2). Seven of the clusters comprised two patients, while the remaining one comprised three patients (total clustered isolates *n* = 17). These eight WGS transmission clusters corresponded to a genomic clustering rate of 11.5%. The remaining 61 (78.2%) isolates were unique and distinct by more than five SNPs from the genetically closest strain. The clustering data demonstrated that 9 of 17 clustered isolates (52.9%) belonged to phylogenetic lineages 4.7 and 4.8 (mainly T; Figure 2). Three clusters were formed by isolates belonging to the Haarlem lineage (4.1.2.1), and one cluster contained two isolates of the Ural lineage (4.2.1) (Figure 2). None of the clustered isolates belonged to lineage 2. Although almost 50% of all clustered isolates showed a different phenotypic resistance profile, all clustered isolates shared the same genotypic resistance (Figure 2).

## Discussion

In this study, we compared the results of pDST with genomic data in 78 mono- and polyresistant TB strains and found that a large proportion of phenotypically resistant isolates (46.1%) do not harbour the gene mutations encoding resistance based on the WHO catalogue. One possible explanation could be either false-positive results of pDST or false-negative results of WGS. Many factors generally affect the implementation and correct interpretation of pDST, including culture contamination, laboratory errors (e.g. human errors during the testing process, such as mislabelling samples, mishandling cultures, or incorrect data entry), incorrect preparation of drug concentrations or using expired drugs, improper preparation inoculum, heteroresistance or microcolony growth.^6^ Therefore, to mitigate false-positive results, it is crucial to maintain strict laboratory protocols, conduct quality control checks, regularly calibrate equipment, and use multiple complementary testing methods. False-positive results can lead to inappropriate treatment regimens for TB patients and can result in the development of MDR TB, highlighting the potential of WGS-based resistance prediction. Although WGS provides more reliable results with respect to pDST, false-negative results can occur, mainly due to the insufficiently described function of mutations involved in the emergence of drug resistance that may occur outside the commonly targeted regions in the *M. tuberculosis* genome and some resistance mechanisms may not yet be well characterized or included in reference databases used for WGS analysis.^17^ Also, WGS primarily focuses on single-nucleotide variations (SNVs) and may miss insertions and deletions (indels), or structural variations that contribute to drug resistance. These can be challenging to detect accurately with standard WGS pipelines.

Our data showed that isoniazid-monoresistant strains are the most prevalent among all forms of DR-TB. The same results were demonstrated in other studies; therefore, attention must be paid to this form of DR-TB to avoid evolving towards MDR-TB.^18,19^ Moreover, none of the isolates showed monoresistance to rifampicin by pDST, indicating the low frequency of these forms of TB in the Czech Republic and Slovakia during the period of 2018–20. These results are supported by previous studies that showed that monoresistance to rifampicin is less frequent compared with monoresistance to isoniazid.^20,21^ However, based on the WGS results, three isoniazid-monoresistant isolates showed genotypic-encoding resistance to rifampicin (high-confidence mutations in the *rpoB* gene: I491P; L430P; L452P), meaning we missed three MDR cases. Furthermore, the resistance encoded by these mutations is not detected by the conventional molecular genetic assays, and therefore only WGS methods allow reliable detection of this mutation.^22,23^

Overall predictive sensitivity and specificity of WGS in our study ranged from 15% and 92.3% to 92.7% and 100%, respectively. For isoniazid and ethambutol, the sensitivity (92.7%/75.0%) and specificity (92.3%/98.6%) of WGS, as well as the most common mutations encoding resistance, are consistent with previous reports.^24,25^ Only three different mutations in three candidate genes (*katG*, *inhA* and *fabG*) and two mutations in one candidate gene (*embB*) were identified for isoniazid and ethambutol, respectively. All these mutations were confirmed to be associated with resistance.^26^

Our analysis showed that phenotypic monoresistance to streptomycin was the most prevalent drug resistance pattern among all isolates. These results are supported by other studies confirming that monoresistance to streptomycin is the most widespread type of *M. tuberculosis* resistance in Western Europe.^27,28^ Moreover, we observed a low sensitivity of WGS (41.9%) in the prediction of streptomycin resistance compared with pDST due to unidentified gene mutations encoding resistance in 25/43 (58.1%; 24/25 belonged to lineage 4) phenotypically resistant strains. Twelve of these isolates showed a reduced growth rate, and 10 isolates exhibited normal growth in a medium containing streptomycin at the CC (4 mg/L), suggesting that this drug may still be effective for TB patients. Three isolates were also resistant to streptomycin at a concentration above the CC (10 mg/L), suggesting unknown genetic bases for streptomycin resistance. By variant-calling analysis, we characterized mutations in *gidB*, *rrs* and *whiB6* genes classified in ‘Group 3: uncertain significance’ in the WHO catalogue in 15/25 (60%) phenotypically resistant isolates with the variable extent of growth in different concentrations of streptomycin (Table 3). These gene variants were reported in previous studies in phenotypically resistant isolates (for example, in codons 34 and 77 of the *gidB* gene), but more data and further evidence are necessary.^29,30^ Also, it was shown that none of these mutations is present simultaneously in both susceptible and resistant isolates of *M. tuberculosis*, which highlights their role in resistance (Table 3). We believe that our results may contribute to reclassifying some of the mutations with uncertain significance into the group of mutations associated with resistance in the WHO catalogue. Also, two novel frameshift mutations in the *gidB* gene (4407847–4407848 Del GC; 4407714 Del T) were identified in two phenotypically resistant isolates. The WHO catalogue contains *gidB* deletions conferring streptomycin resistance at positions 4407846, 4407847, 4407849, 4407850 and 4407713; therefore, we assume that novel deletions identified in our study could be associated with resistance due to their proximity, but further studies are necessary. These data indicate that the discrepancies between pDST and WGS are primarily caused by the difficulty in interpreting the pDST results and the incomplete library of characterized genomic mutations encoding resistance to streptomycin. We also investigated the association between phylogenetic lineage and genotypic resistance to streptomycin and found that most isolates (88.2%) belonged to lineage 2.2.1 and lineage LAM 4.3 (Figure 1). Similar observations have also been described in an earlier study conducted outside Europe.^31^

The overall sensitivity and specificity of WGS in the prediction of pyrazinamide resistance were only 15% and 94.5%, respectively. We detected a mutation conferring resistance (*pncA* A134V, *pncA* W119C) in 1 of 18 phenotypically resistant isolates and in 4 phenotypically susceptible isolates. This is primarily due to challenges faced with pyrazinamide pDST using the MGIT 960 system, including difficulties in maintaining pH as a result of high inoculums, leading to false resistance or failure of strains to grow at this pH, and resistance mutations do not feature in the catalogues used for the interpretation of variants.^32^ We found variants in the *kefB* (locus Rv3236) gene in two pyrazinamide-monoresistant isolates. A previous study showed that integral membrane transport protein KefB is a K^+^/H^+^ antiporter that releases K^+^ to the phagosomal space and prevents its acidification; therefore, it can play a crucial role in resistance to pyrazinamide.^33^ Two pyrazinamide-resistant isolates had a mutation in *pncA* at codon 87, similar to other studies.^34^ This mutation is classified in the catalogue of mutations published by WHO in ‘Group 5: not associated with resistance’ to pyrazinamide, but its role in resistance should be re-evaluated. Furthermore, 94.5% of the phenotypically pyrazinamide-resistant isolates belong to lineage 4, and these findings are consistent with those reported elsewhere.^35^ Based on previous studies emphasizing the importance of retesting drug susceptibility to pyrazinamide, we repeated the pDST to pyrazinamide, which significantly increased the sensitivity of WGS (Table 2). The same rate of false phenotypic resistance to pyrazinamide after repeated testing was also shown in another study, which highlights the utility of WGS.^36^ Repeated pDST increases costs for clinical laboratories, while the decreasing costs of WGS and next-generation sequencing (NGS) technologies offer the potential for their application in routine practice to provide clinicians with even more detailed and timely results.^37^

For other second-line anti-TB drugs, including bedaquiline and delamanid, all isolates were phenotypically susceptible to all tested concentrations (Table 2). Genotypic data revealed several mutations with uncertain significance in resistance to these drugs, which may help further evaluate the role of genotypic data in resistance.

In our study, phylogenetic analysis showed a variable representation of mono- and polyresistant isolates within phylogenetic lineages. We found that the Haarlem lineage (4.1.2.1) and mainly the T lineage (4.7; 4.8) were the predominant lineages; however, in 60.4% of isolates, a mutation conferring resistance to at least one drug was not identified. These results are similar to a previous finding that reported lineage 4 as the most frequent among drug-susceptible strains of *M. tuberculosis* in European countries.^38^ Lineage 2 included 14 isolates (17.9%), of which 13 (92.9%) showed genotypic resistance to at least one antituberculosis drug (primarily to streptomycin and isoniazid). This supports the theory that higher mutation rates of *M. tuberculosis* Beijing strains during human infection are likely to account for the higher adaptability and rapid acquisition of drug resistance compared with lineage 4.^39^ Also, the phylogenetic data were consistent with previous reports that confirmed the spread of lineage 4 and 2, mainly in Europe and Eurasia.^40,41^

The percentage of genomically linked cases (≤5 SNPs) was 21.8%. The clustering rate for isolates in this study is noticeably lower than in previous reports in different countries within Europe.^38^ This may be due to false resistance based on pDST, as transmission chains may have been present within phenotypically susceptible isolates, but these were not included in our study. Also, the high frequency of drug resistance among isolates with no close genetic relationship with other isolates reflects development through selective pressure, in which a small population of bacteria genetically mutate to provide drug resistance. Our results demonstrated that all clustered isolates belonged to Euro-American lineage 4. The clustering rate among Beijing strains was null, which is in contrast to other studies that have confirmed increased transmissibility of strains of this lineage.^31,42^ It can be explained by the fact that many of the patients infected by Beijing *M. tuberculosis* strains come from countries outside the Czech Republic, thus, we miss many isolates, which might lead to misinterpretation of the transmission. We expect a significant increase in resistant strains of the Beijing lineage due to massive migration events related to the war in Ukraine, which will be the subject of our further research.

In conclusion, our study showed the difficulty of pDST interpretation and a high proportion of false-positive resistance compared with WGS. We repeated the pDST to confirm the role of novel mutations in resistance, but more samples are still needed, representing a limit of our study. Our results confirmed that WGS technology is a method that provides clinicians with more accurate and more informative results compared with conventionally used methods, but pDST for some drugs is still needed to confirm susceptibility and construct the final regimens. Also, we suggest conducting either WGS or targeted NGS to confirm drug resistance in TB cases that initially test positive via phenotypic or rapid genetic drug testing and thus mitigate the risk of false positives. Nevertheless, additional research endeavours aimed at deciphering the mechanisms and mutations linked to bacterial drug resistance, particularly through mycobacterial genome-wide association studies (mGWAS), are imperative to fully harness the capabilities of sequencing technologies. In addition, the data showed that the development of resistance occurs mainly through selective pressure and not by direct transmission of resistant strains between patients.

## Supplementary Material

dlad108_Supplementary_Data

## Data Availability

WGS raw reads were submitted to the National Center for Biotechnology Information as FASTQ files under study Accession No. PRJNA886608.

## Supplementary data

Figure S1 is available as Supplementary data at *JAC-AMR* Online.
