# Supplementary material for: Resistance patterns and transmission of mono- and polyresistant TB: clinical impact of WGS
Source: JAC Antimicrob Resist. 2023 Oct 4;5(5):dlad108. doi: 10.1093/jacamr/dlad108 (PMC10549209; doi:10.1093/jacamr/dlad108)
Supplement: dlad108_Supplementary_Data [file dlad108_supplementary_data.docx]

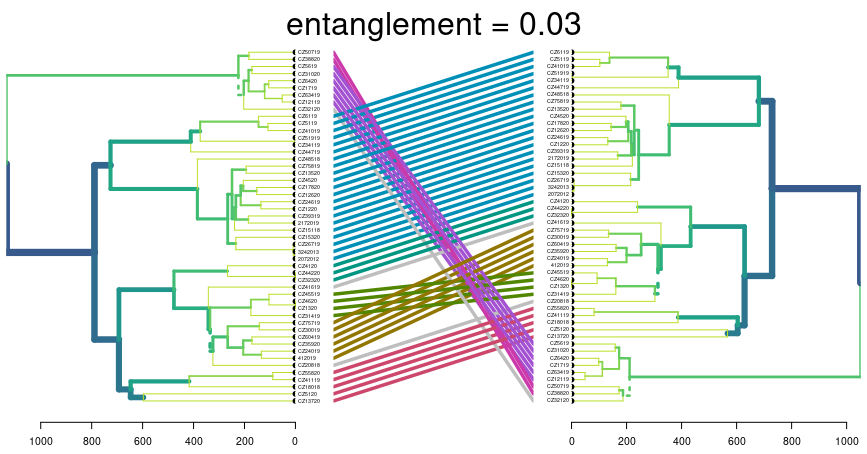


**Supplementary Figure 1.** Tanglegram to compare 50 samples with high coverage and the 50 samples within the 78 samples. The plot illustrating that even considering the strains with lowest coverage, the phylogeny tree is not affected.
